# Invasive earthworms shift soil microbial community structure in northern North American forest ecosystems

**DOI:** 10.1016/j.isci.2024.108889

**Published:** 2024-01-12

**Authors:** Olga Ferlian, Kezia Goldmann, Michael Bonkowski, Kenneth Dumack, Tesfaye Wubet, Nico Eisenhauer

**Affiliations:** 1 German Centre for Integrative Biodiversity Research (iDiv) Halle-Jena-Leipzig, Puschstrasse 4, 04103 Leipzig, Germany; 2 Institute of Biology, Leipzig University, Puschstrasse 4, 04103 Leipzig, Germany; 3 UFZ-Helmholtz Centre for Environmental Research, Department of Soil Ecology, Theodor-Lieser-Str. 4, 06120 Halle (Saale), Germany; 4 Terrestrial Ecology, Institute of Zoology, University of Cologne, Zülpicher Str. 47b, 50674 Köln, Germany; 5 UFZ-Helmholtz Centre for Environmental Research, Department of Community Ecology, Theodor-Lieser-Str. 4, 06120 Halle (Saale), Germany

**Keywords:** Ecology, Microbiology, Soil ecology

## Abstract

Invasive earthworms colonize ecosystems around the globe. Compared to other species’ invasions, earthworm invasions have received little attention. Previous studies indicated their tremendous effects on resident soil biota representing a major part of the terrestrial biodiversity. We investigated effects of earthworm invasion on soil microbial communities in three forests in North America by conducting DNA sequencing of soil bacteria, fungi, and protists in two soil depths. Our study shows that microbial diversity was lower in highly invaded forest areas. While bacterial diversity was strongly affected compared to fungi and protists, fungal community composition and family dominance were strongly affected compared to bacteria and protists. We found most species specialized on invasion in fungi, mainly represented by saprotrophs. Comparably, few protist species, mostly bacterivorous, were specialized on invasion. As one of the first observational studies, we investigated earthworm invasion on three kingdoms showing distinct taxa- and trophic level-specific responses to earthworm invasion.

## Highlights

• Invasive earthworms colonize ecosystems worldwide but received little attention
• We investigated effects of earthworm invasion on soil bacteria, fungi, and protists
• We found an overall negative effect of earthworm invasion on soil microorganisms
• Fungal diversity was relatively robust to invasion, but species composition changed

## Graphical abstract

**Figure:**
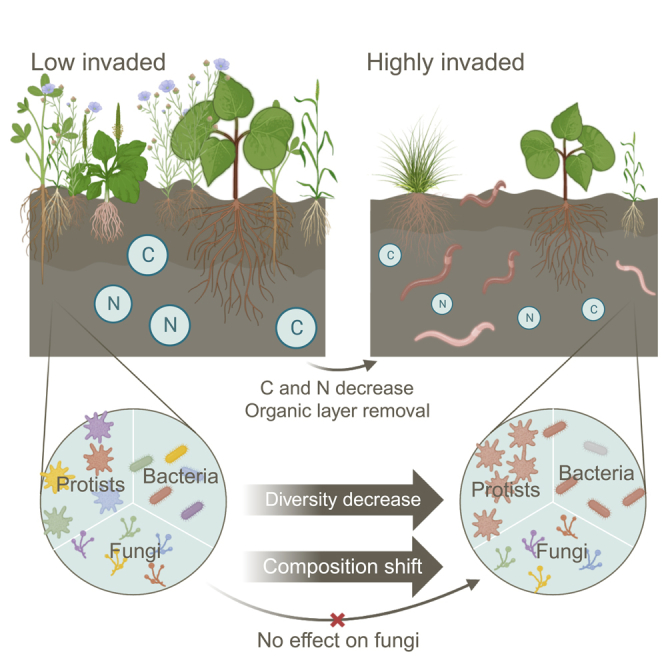

## Introduction

Species invasion is among the most dominant global change drivers threatening native biodiversity and ecosystem properties.^1^^,^^2^^,^^3^^,^^4^ Invasion success and its impacts are known to be greatest when invaders have functionally distinct attributes to the recipient community and, thus, occupy empty trophic niches with largely unlimited resources.^5^ This has often been found for invaders in soil, such as invasive earthworms, which colonize ecosystems around the globe.^6^^,^^7^^,^^8^ However, compared to other species invasions, such as that of large mammals and plants, invasive earthworms have received comparably little attention in terms of specific measures to avoid or mitigate their invasion.^9^ This still reflects the strong discrepancy in knowledge between the above-vs. belowground system as well as vertebrate vs. invertebrate species.^10^

Earthworm invasion has been constantly facilitated by human activities, such as global trading and angling activities.^8^^,^^11^ Its causes and consequences are particularly well-studied in northern North American forests, where large parts have been devoid of native species since the last glaciation.^12^ Native macro-detritivores in these forests are virtually lacking here. In their role as invaders, earthworms cause substantial alterations to their local environment^11^^,^^13^^,^^14^ and, thus, notably shape ecosystem functioning.^15^^,^^16^^,^^17^ They create dense networks of burrows, mix soil layers and, subsequently, redistribute nutrients among horizons, secrete nutrient-rich compounds in the form of mucus and casts, and remove substantial parts of the leaf litter.^13^^,^^18^^,^^19^ These activities interactively increase important soil processes, such as water infiltration, nutrient mineralization, carbon stabilization, and litter decomposition rates.^20^ Likewise, it is not surprising that earthworms have been found to tremendously affect the resident faunal, plant, and soil microbial communities that either drive or depend on these processes.^14^^,^^21^^,^^22^^,^^23^

Earthworm invasion has been particularly shown to shift the composition and functionality of soil microbial communities.^23^^,^^24^ Thereby, different underlying mechanisms act on different microbial groups,^25^^,^^26^ e.g., earthworms facilitate access to remotely available resources for bacteria due to the break-down of organic matter.^22^^,^^27^^,^^28^ However, endogeic earthworm species that feed in mineral soil may also diminish carbon availability to bacteria due to the formation of recalcitrant organo-mineral complexes via gut passage.^29^^,^^30^ But among the microbial groups, earthworm effects on bacteria have also been found to be comparably weak.^22^ Similarly, effects of invasive earthworms on protist communities can be positive as well as negative: earthworms are known to foster protist cyst distribution through ingestion,^31^ but at the same time, active forms can serve as food source for earthworms.^32^ Moreover, due to the fact that the majority of protist species has been considered to live on a bacterial-dominated diet, it can be assumed that impacts follow changes in bacterial communities.^33^^,^^34^ In contrast, as recently shown, effects on soil fungal growth are rather consistently negative compared to the former two groups.^22^ Through their burrowing activities, earthworms remove fungal-rich organic layers, disrupt fungal hyphae, and also directly feed on fungi whose larger-sized structures are more sensitive to gut passage than that of bacteria.^26^^,^^35^ Overall, with earthworm invasion, a shift from a fungal to a bacterial biomass-dominated system was suggested.^26^^,^^36^^,^^37^ However, the impacts may differ across fungal taxa due to different life strategies.^25^ As suggested for plants in ecosystems under earthworm invasion, microbial communities may pass through a novel environmental trait filter that selects for specific taxa, functional roles, and feeding types being typically diverse in fungi and protists.^34^^,^^38^^,^^39^ However, such functional roles have been rarely identified for different microbial groups at the same time, especially for protists, under the impact of earthworm invasion.

Previous studies on the effects of invasive earthworms on soil biota further revealed the importance of function of soil depth.^22^ Similar to the lack of resolution in microbial taxa, studies neglecting stratification may have masked the contrasting responses of soil biota in organic and mineral layers. Indeed, abundances of different microbial groups follow different gradients with soil depth.^40^ Whereas bacteria preferably utilize resources in mineral soil, fungi exploit resources in organic soil.^26^ In addition, invasive earthworms transport mineral material with less microbial resources upward to organic layers, and introduce substrates rich in organic matter from upper down to mineral layers.^22^^,^^24^ However, we still lack an overarching understanding of the individual responses of microbial groups, such as bacteria, fungi, and protists, to earthworm invasion in different soil layers.

Here, we investigated the effects of earthworm invasion on soil microbial communities comparing areas of low and high earthworm invasion within northern North American forests. For this, we calculated different microbial diversity metrics and assessed community structure based on DNA-sequencing data of soil bacteria, fungi, and protists in two soil depths in two forests in Alberta, Canada, and one forest in Minnesota, USA. We also included environmental properties of the three sites to explore the underlying mechanisms. Further, we conducted an indicator species analysis using the different predictor variables (invasion stage, soil depth, and forest site) to identify specialized microbial species and trophic groups in the three groups that only occur in specific variable levels. We hypothesized that (1) microbial diversity in highly invaded forest areas is lower in the upper soil layer and higher in the lower soil layer. Microbial resources in the upper layer are diluted by the upward transport of mineral material while the lower layer gets enriched by the downward transport of organic matter. While earthworm effects on fungi are considerably strong, effects on bacteria and protists are rather neutral or ambiguous; (2) microbial community compositions differ with forest, invasion stage, and soil depth, but compositions in the two depths are more similar in areas of high than low invasion due to higher bioturbation and, thus, higher resource homogenization among soil layers, compared to those with low earthworm invasion; (3) we further hypothesized that certain microbial species and functional groups are indicative of earthworm invasion, but compared to bacteria and protist communities, fungal communities have more specialized species^41^ that only co-occur in earthworm-free habitats and, thus, serve as indicator species; (4) we find more indicator species for forests and invasion stages than for soil depth, since the specific soil properties in the forests as well as invasive earthworms put a stronger environmental filter on the communities.

## Results

Earthworm abundances in the three forests were 26.8 and 159.5 individuals per m^2^ in the low and highly invaded areas, respectively. Among the forests, Barrier North had the lowest abundances in the low invaded area and the highest contrast between the low and highly invaded areas (Barrier North: 7.2 vs. 98 individuals per m^2^, Barrier South: 40.8 vs. 148.8 individuals per m^2^, St. John’s: 32.4 vs. 231.6 individuals per m^2^; Figure S1).

### Microbial diversity

**Figure 1:**
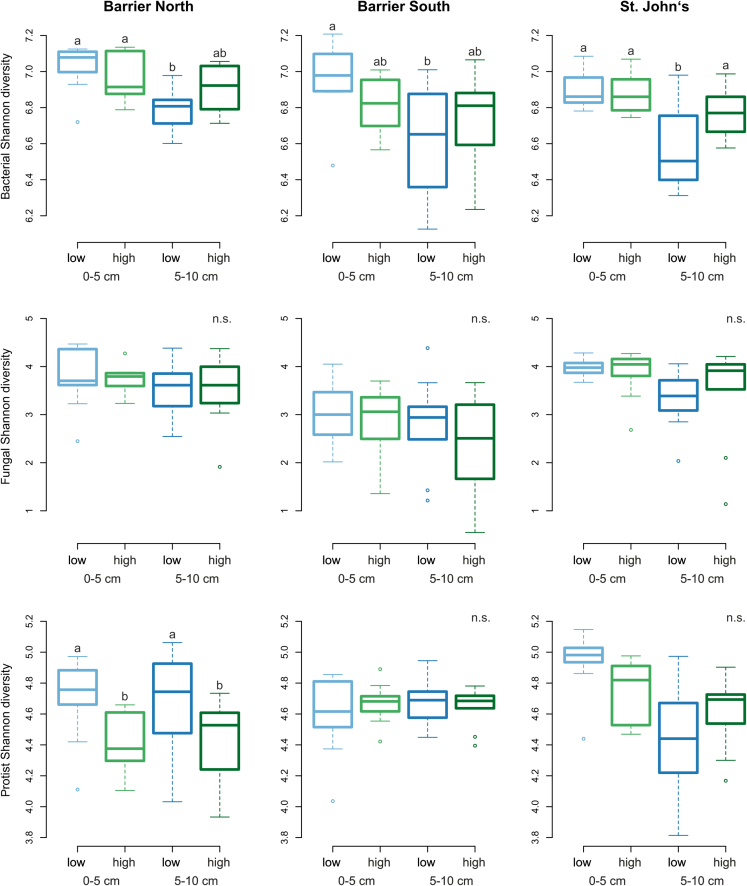
Microbial Shannon diversity index The microbial Shannon diversity index was affected by earthworm invasion stage, forest, and soil depth. Different letters above boxplots indicate significant differences (p ≤ 0.05) according to Wilcoxon rank sum exact test; n.s. – no significant differences between considered predictor variables. See also Figure S2.

Based on the rarefied datasets, a total of 16,390 bacterial ASVs, 2,692 fungal ASVs, and 555 protist OTUs were detected in the 120 soil samples. The Shannon diversity index was overall highest for bacteria and lowest for fungi (Figure 1). The bacterial Shannon diversity index was higher in the upper compared to the lower soil layer, but this effect was only significant in the low invaded area. Most of the differences in the bacterial Shannon diversity index regarding the forests and invasion stage were only non-significant trends. Only in St. John’s, we found a higher Shannon diversity index in the highly invaded area in the lower soil layer. In general, there was a slight pattern of lower microbial diversity in the upper and higher diversity in the lower soil layer in the highly invaded area, respectively. The fungal Shannon diversity index differed neither between soil depth nor invasion stage. However, Barrier South was less diverse in soil fungi than the other two forests (Figure 1). The protist Shannon diversity index appeared to be forest-specific. Whereas in Barrier North similar patterns were found as for the bacterial Shannon diversity index, i.e., higher diversity in the upper soil layer, independent of invasion stage, the protistan Shannon diversity index was similar in all considered predictor variables in Barrier South. In St. John’s, the protistan Shannon diversity index was highest in the upper soil layer with low earthworm invasion, and lowest in the lower soil layer also with low earthworm invasion (Figure 1). Effects in the other metrics of microbial diversity were similar and can be found in Figures S2–S4.

### Environmental parameters

In two of the three forests, the humus layer was significantly lower in highly compared to low invaded areas (Barrier North: F_1,18_ = 6.42, p = 0.02; Barrier South: F_1,18_ = 0.01, p = 0.92; St. John’s: F_1,18_ = 78.76, p = < 0.0001; Table S1), whereas litter weight was not significantly affected. Further, in all forests, soil nitrogen (Barrier North: F_1,18_ = 35.90, p < 0.0001; Barrier South: F_1,18_ = 37.72, p < 0.0001; St. John’s: F_1,18_ = 40.58, p = < 0.0001) and carbon content (Barrier North: F_1,18_ = 35.55, p < 0.0001; Barrier South: F_1,18_ = 41.89, p < 0.0001; St. John’s: F_1,18_ = 35.08, p = < 0.0001) as well as microbial biomass (Barrier North: F_1,18_ = 33.45, p < 0.0001; Barrier South: F_1,18_ = 58.26, p < 0.0001; St. John’s: F_1,18_ = 20.06, p = < 0.001) in the upper soil layer were significantly lower in highly compared to low invaded areas. In the lower soil layer, this effect was only present in Barrier South (nitrogen: F_1,18_ = 26.64, p < 0.0001; carbon: F_1,18_ = 23.94, p < 0.001; microbial biomass: F_1,18_ = 42.34, p = < 0.0001).

Interaction effects of invasion stage and environmental variables on the microbial Shannon diversity index were significant for litter weight and carbon content in bacteria in the lower soil layer (F_1,18_ = 9.63, p = 0.02 and F_1,18_ = 17.15, p = 0.01, respectively; Table S2); for litter weight, nitrogen content, and microbial biomass in fungi in the upper soil layer (F _1,18_ = 14.37, p = 0.03; F_1,18_ = 59.92, p = 0.004; F_1,18_ = 25.59, p = 0.02, respectively); for carbon and nitrogen content in fungi in the lower soil layer (F_1,18_ = 315.83, p = 0.04 and F_1,18_ = 247.84, p = 0.04, respectively); and for humus layer in protists in the upper soil layer (F_1,18_ = 10.67, p = 0.05). However, environmental variables like the thickness of the humus layer and pH did not interact with earthworm invasion stage in any of the microbial groups, forests, nor soil depths.

### Microbial community composition

**Figure 2:**
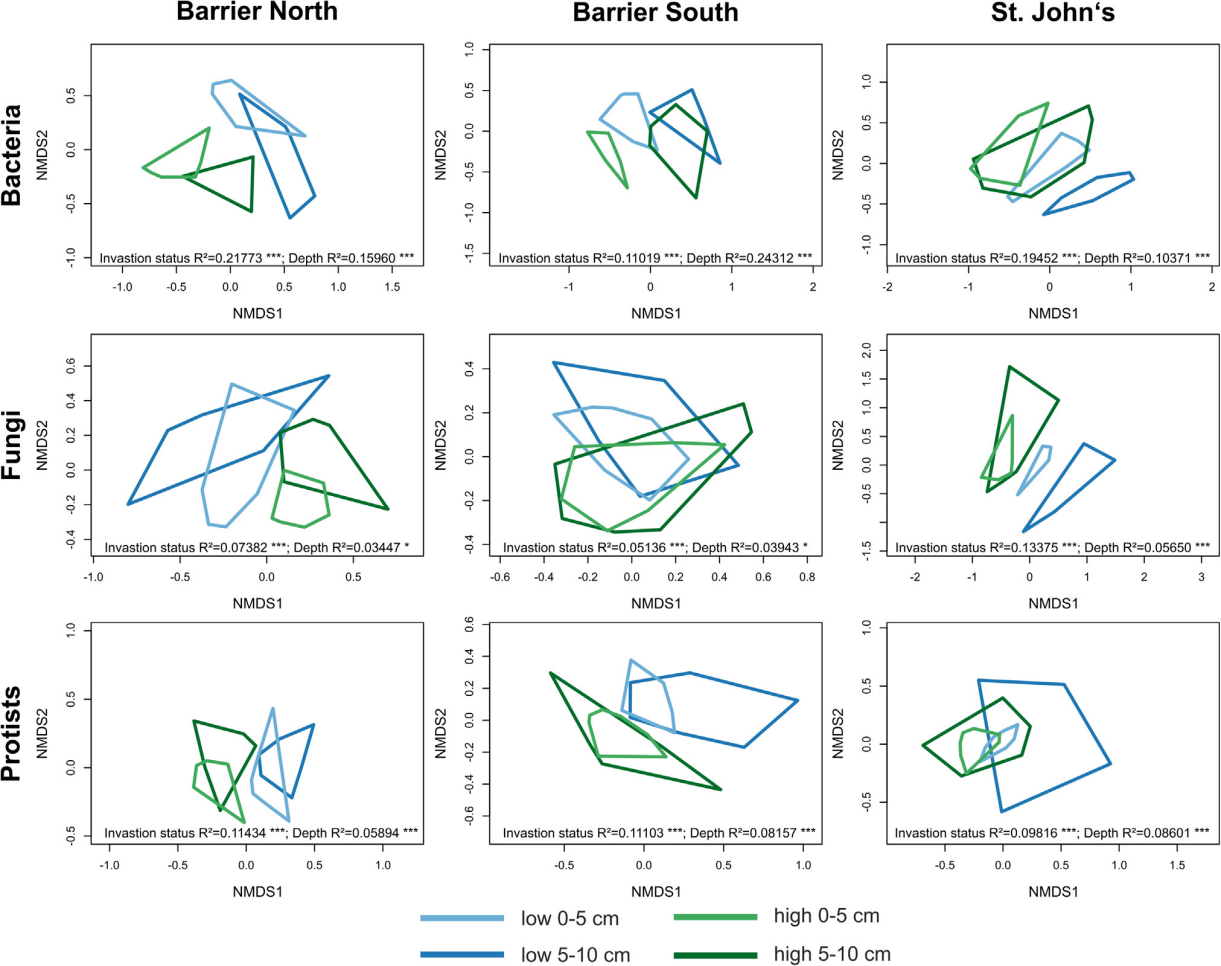
Microbial community composition Non-metric dimensional scaling displaying the microbial community dissimilarity, based on Bray-Curtis distances was affected by earthworm invasion stage and soil depth for each forest individually; perMANOVA results, i.e., R^2^ value and p values: ∗ - p ≤ 0.05, ∗∗ - p ≤ 0.005; ∗∗∗ - p ≤ 0.001, are displayed in the individual panels.

Microbial communities differed significantly between the two forests in Canada (North and South Barrier) and St. John’s in the USA (perMANOVA: bacteria – R^2^ = 0.2556, p = 0.001; fungi – R^2^ = 0.1350, p = 0.001; protists – R^2^ = 0.1482, p = 0.001; Figure 2). Considering the forests individually, both invasion stage and soil depth shaped the microbial communities significantly. But fungi displayed rather weak differences with soil depth in the two Canadian forests. In most of the forests and microbial groups, communities were more similar among soil depths than among earthworm invasion stages. In John’s, communities were more similar among depths in the highly compared to the low invaded area. In the upper soil layer, community compositions had generally lower variation across invasion stages, as indicated by the width of the convex hulls.

**Figure 3:**
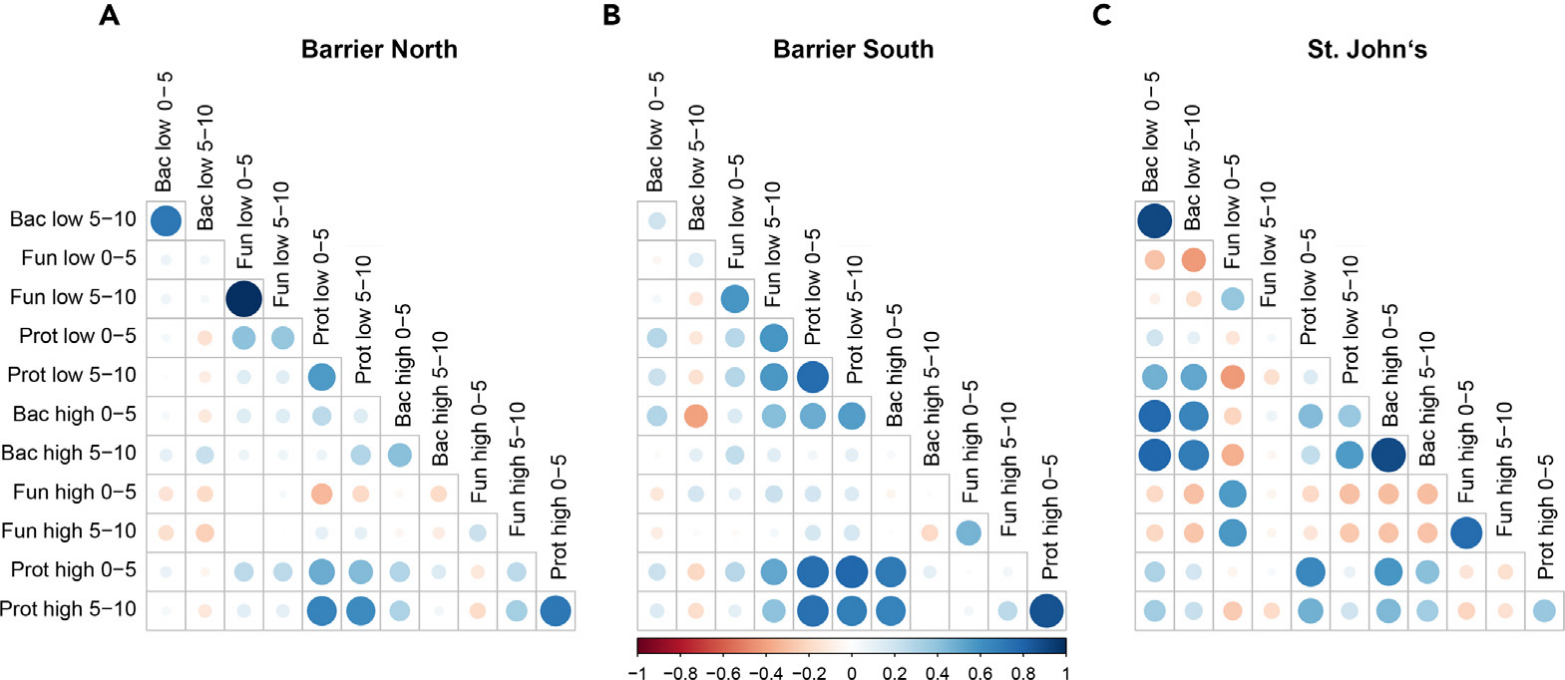
Mantel correlations displaying the intra- and inter-kingdom relationships Correlations among community dissimilarities as affected by invasion stage and soil depth, between the individual forest-specific microbial community compositions based on Bray-Curtis distance matrices for bacteria, fungi, and protists for (A) Barrier North, (B) Barrier South and (C) St. John’s; circle size represents Mantel R correlation values with blue circles representing positive and red circles negative correlations. Absence of a circle indicates no significant correlation.

Mantel tests, performed to test for correlation strengths among individual microbial community dissimilarities (Figure 3), showed that the strongest correlations were within-kingdom, e.g., bacterial community dissimilarities were highly correlated across depths in the low invaded area at Barrier North (Figure 3A) and St. John’s (Figure 3C), and among soil depths compared to invasion stages. Barrier North showed the lowest number of correlations, St. John’s the highest. The latter had the most negative correlations.

### Microbial taxonomic composition and indicator species

**Figure 4:**
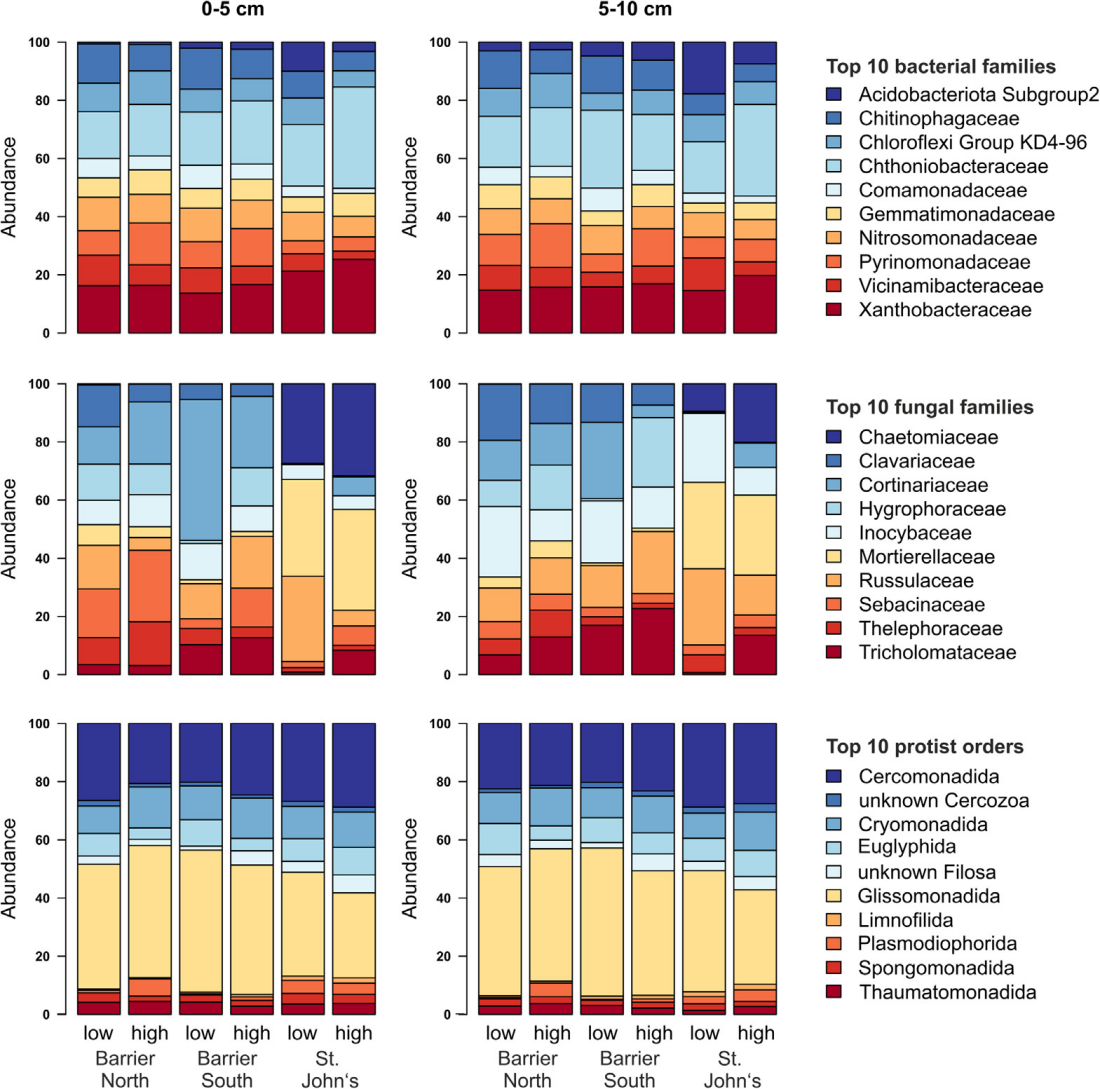
Relative abundances of microbial taxa Relative abundances of the top ten bacterial and fungal families and protist orders, respectively, as affected by invasion stage and forest for the two considered soil depths.

The evaluation of the most abundant microbial families/orders showed forest-specific taxa, e.g., the fungal Chaetomiaceae occurring only in St. John’s. It further showed that groups like the bacterial Chitinophagaceae or the protistan Glissomonadida were more abundant in the low compared to the highly invaded area. Interestingly, Chitinophagaceae were previously reported to positively correlate with earthworm abundances due to higher nitrogen availability.^63^ In our study, however, nitrogen contents were lower in the highly invaded area which again corresponds with the effects in Chitinophagaceae. The protistan Plasmodiophorida and fungal Sebacinaceae, for instance, were more abundant in the highly invaded area. The other groups displayed no clear pattern of preferences (Figure 4). In general, fungi responded more sensitively to all predictor variables compared to the other two microbial groups. All groups did not differ strongly between soil depths.

The identification of the most important indicator ASVs and OTUs, respectively, showed that irrespective of the predictor variable, fungi had the highest number of indicator species, followed by bacteria, and protists (proportions of total ASV and OTU numbers; Table S3). Among the three predictor variables, forest had the highest number of indicator species, followed by invasion stage, and soil depth. The results showed that more bacterial, fungal, and protist taxa were potentially indicative for St. John’s in comparison with the forests in Canada, i.e., North and South Barrier. Likewise, there were more indicator species identified for the low invaded area and the upper soil layer. Most of the bacterial indicator species were chemoheterotrophic, but for roughly half of the bacterial families, no information on functions was available. Further, we did not find any differences in function among the levels of the three predictor variables forest, invasion stage, and soil depth. Most of the fungal indicator species were saprotrophic. However, in the forests, those were interspersed with ectomycorrhizal fungi in Barrier North and plant pathogens in St. John’s. The other predictor variables did not differ in terms of dominance of trophic groups. Trophic groups of protist indicator species did not differ between predictor variable levels. All three forests had a mix of bacterivores and omnivores, and in the two invasion stages, bacterivores dominated. For soil depth, we only found a few indicator species that were mostly bacterivores.

## Discussion

In their role as ecosystem engineers, earthworms may cause tremendous shifts in ecosystems devoid of native species or functionally similar taxa.^5^ Yet, the specific effects on soil microbial communities and their functional roles have received little attention in this context. Our study is, to our knowledge, one of the first observational studies investigating earthworm invasion effects on three kingdoms at the same time, i.e., bacteria, fungi, and protists, using DNA metabarcoding. We aimed at broadening our understanding of the impact of invasive earthworms on the community composition and trophic groups of three microbial kingdoms. Moreover, we explored two soil depths as well as different forest types to see how generalizable our results are.

### Invasion effects on microbial diversity

Earthworm abundances in the low invaded area were considerably lower (close to zero) than in the highly invaded area in all three forests. This confirms our selection of the respective sampling points according to the invasion stages of low and high earthworm invasion. In comparison to the other two forests, in Barrier North, the low invaded area had the lowest earthworm abundance, and the contrast between low and highly invaded was most pronounced. This suggests that the invasion front in Barrier North is much clearer, and/or the selected sampling points were more distant from the invasion front.

The Shannon diversity index was highest for bacteria and low in protists and fungi which is in line with a multitude of ecosystems.^64^^,^^65^ Generally, highly invaded areas had lower diversity in all three microbial groups and a generally lower soil microbial biomass. This coincides well with the lower nitrogen and carbon contents we found in the highly invaded area. However, it has to be taken into consideration that we cannot infer causal relationships, i.e., microorganisms rely on these elements as resources, but also and more importantly, microorganisms also determine nutrient and carbon cycling.^66^ The effects we found were partly weak as well as slightly different across the groups, i.e., they were minor for bacterial diversity (trends, only significant in St. John’s at 5–10 cm), non-significant for fungal diversity, and well-pronounced for protists (in Barrier North). The same patterns were largely observed for the other diversity metrics measured. Compared to bacteria, fungi were not affected by earthworm invasion, which was in contrast to our hypothesis. However, as known oligotrophs, they may be more tolerant to environmental stress due to their ability to utilize diverse resources.^67^ This results in a broader resource spectrum and a potential for resource shift with earthworm invasion. Interestingly, former studies, conducted at both the present and other sites, showed significant positive as well as negative effects on fungi and a shift from fungal to bacterial biomass-dominated communities with earthworm invasion.^22^^,^^36^^,^^68^ However, evidence sometimes comes from studies that investigated other habitats as well as microbial abundance and biomass and not diversity. Different mechanisms may underlie the contrasting effects of these measures under environmental stress.^69^^,^^70^ Furthermore, the dominant fungal families were mainly saprotrophic, which are typically less disturbed by earthworm bioturbation activities compared to mycorrhizal fungi that rely on sensitive hyphal networks.

Within the large pool of earthworm-facilitated soil processes, invasive earthworms can have both positive and negative effects on the resident microbial communities.^7^ Depending on the habitat and on the experimental system (field observation vs. field experiment vs. lab experiment), different drivers may dominate.^14^^,^^71^ It is conceivable that processes stabilizing organic material in the form of organo-mineral complexes may have played a larger role in our studied forests than that mobilizing resources. This had negative effects on bacterial (labile resource uptake) but no effects on fungal diversity (labile and recalcitrant resource uptake). Among the microbial groups, protistan diversity was most severely affected by earthworm invasion. Protist communities include a large proportion of species on higher trophic levels, such as bacterivores, eukaryvores, and omnivores.^34^ Given that bacteria are a food source for most of the protist species, protistan diversity patterns may have resembled that of bacteria. The effects may have even been stronger for protists, because the impact of environmental stress on consumers is assumed to increase with trophic level.^72^

We found some significant interactions between earthworm invasion and measured environmental variables that point to an influence of carbon and nitrogen contents, and organic layer depth on the effects of invasion on bacterial and fungal diversities. This suggests that the severity of the earthworm invasion on the resident microbial community depends to a certain extent on the abiotic prerequisites of the habitat.

### Invasion effects on microbial community composition

Soil microbial community composition differed between the three forests, soil depths, and earthworm invasion variables in all three microbial groups. However, community compositions differed more between earthworm invasion stages than between soil depths, suggesting that earthworms pose a stronger environmental filter on soil microbiota than soil-property gradients along the soil profile. Moreover, Mantel tests indicated the most significant pairwise (positive and negative) correlations between predictor variables and microbial groups in St. John’s compared to the other two forests. This points to a pronounced site effect, i.e., forests and their main vegetation and ecosystem properties shape soil microbial communities and their relatedness, which is also in line with the environmental properties of the sites, such as the distinct carbon and nitrogen contents.^73^

Assessments of the ten dominant taxa for the three microbial groups revealed that the saprotrophic fungal Chaetomiaceae were only found in St. John’s. This site was considerably different from the other two forest sites in terms of plant communities, environmental properties, and soil type that may have led to distinct microbial taxa.^45^^,^^74^ Furthermore, the protistan Plasmodiophorida were positively affected by earthworm invasion. Plasmodiophorida is a group of endoparasitic species in plants, algae, and fungi. It can be assumed that, under earthworm invasion, certain host taxa were impaired, and, therefore, their susceptibility to pathogen infestations was higher. Overall, compared to bacteria and protists, fungal family dominance was most severely shifted with earthworm invasion and across forests. In contrast, bacterial and protist diversity and species compositions were affected by earthworm invasion but rather within family/order level. The fact that fungi are relatively species-poor, at least in comparison to bacteria, may have contributed to these contrasting effects.

### Differences with soil depth

Earthworm invasion can change soil abiotic properties as well as shift faunal and microbial communities as shown before.^14^^,^^22^ These studies also demonstrated that it is essential to consider soil stratification. Earthworm effects were even found to be opposed in different soil layers, i.e., negative in upper and positive in lower soil layers, that sometimes led to a neutral overall effect masking these specific responses and mechanisms at different soil depths. We, therefore, included soil depth as a predictor variable in all analyses. Overall, we observed that the diversity of microorganisms decreased with soil depth, especially in the area of low earthworm invasion, but the dominance of families/orders did not change. The proportion of nutrients and number and size of water-filled soil pores typically decrease with soil depth and, consequently, the amount of microbial resources and habitats, which restricts microbial communities to a limited species spectrum.^75^^,^^76^ In our study, nitrogen and carbon contents in the two soil layers also consistently decreased with soil depth. However, these shifts in microbial communities with depth acted relatively evenly on the different families/orders. In addition, variation in microbial community composition increased with soil depth. Soil faunal diversity and abundance that is responsible for the major part of the bioturbation activity in soil also decreases with depth.^77^^,^^78^ Lower bioturbation may, in turn, lead to higher heterogeneity of microbial resources which results in the high variation of microbial community composition that we found in lower soil layers. Interestingly, such a microbial community shift with soil depth was specifically pronounced in the low invaded areas suggesting that earthworms may disrupt the above explained gradients of abiotic and biotic conditions.

We hypothesized that earthworm invasion has opposing effects on microbial groups in the upper and lower soil layers, as microbial resources in upper layers are diluted by the upward transport of mineral material and that in the lower layers are enriched through the downward transport of organic matter.^14^^,^^22^ Despite a respective general trend for this pattern, our results were mostly not significant and only give weak support to this hypothesis. These findings may be linked to the weak patterns we found in soil resources, such as nitrogen and carbon content, that were lower in the highly invaded area compared to the low invaded area in the upper soil layer. However, we found only weak or no effects in the lower soil layer. Nevertheless, among the microbial groups, bacteria showed the strongest effects and fungi the weakest, which is in line with our findings. Fungal diversity and community composition were relatively robust to earthworm invasion as well as soil depth which again points to their ability to utilize rather complex and recalcitrant resources compared to other microbiota resulting in a broader resource spectrum.^67^^,^^79^ Furthermore, fungal networks contribute to resource supply on a larger spatial scale than bacteria do.^80^

Due to the fact that earthworms considerably bioturbate the soil,^18^ we assumed that earthworm invasion leads to a higher mixing of soil layers which is reflected in more similar microbial community compositions between layers in highly invaded areas and more distinct compositions in low invaded areas.^81^ However, we only found this pattern in St. John’s for all microbial groups. This is rather surprising as this forest had the lowest contrast in earthworm abundance among the forests which suggests that the bioturbation effects would be comparably low. But this forest also had the largest distance between the low and highly invaded area, and other factors than earthworm presence may have distinguished them and contributed to a vertically homogeneous and heterogeneous microbial community in the low and highly invaded areas, respectively.^45^ This calls for controlled field experiments that standardize all environmental factors and only manipulate earthworm invasion stages.^37^

### Importance of microbial indicator species and functional groups

Among the three predictor variables tested in the study (forest, invasion stage, and soil depth), we found the most microbial indicator species in the three forests. Among these, the most indicator species were found in St. John’s where the plant community considerably differed from these of the other two forests. We found an intermediate number of indicator species in the invasion stages. The lowest numbers were found in the soil depth variable. It is remarkable that the above-mentioned patterns were the same in all three microbial groups. Our hypothesis that such indicator species exist for low and highly invaded areas of the forest was confirmed. Particular species were more indicative for forest and invasion stage than for soil depth confirming our hypothesis. In other words, those communities were more distinct among the first two variables, whereas similar microbial species could be found in different soil depths. Forest and earthworm invasion seemed to have placed a stronger environmental filter on the microbial communities.^37^^,^^38^ This finding supports our comparisons of microbial community compositions. Further, we found more indicator species for the low compared to the highly invaded areas. This suggests that more microbial species can cope with environmental properties unaffected than affected by earthworms which corresponds with the microbial diversities found. We hypothesized that fungal communities have more specialized species than the other two groups^41^ and found, indeed, numbers of indicator species decreasing in the order fungi, bacteria, and protists, confirming our hypothesis. On the other hand, fungal diversity was not significantly affected by earthworm invasion, suggesting a species turnover without a change in diversity.

We assigned functions (trophic groups) to the families/orders of the dominant indicator species in the three microbial groups. Most of the bacterial indicator species were aerobic chemoheterotrophic, and we did not find any effects. Aerobic chemoheterotrophs form one of the largest groups within the bacterial kingdom, which are capable of coping with different environments and disturbances, such as earthworm invasion.^82^

Most of the fungal indicator species were saprotrophic which is not surprising, since the majority of fungal species has a saprotrophic strategy. However, among the indicator species for the three forests, we found ectomycorrhizal species in Barrier South and plant pathogenic species in St. John’s in addition to saprotrophs. Plant communities in Barrier South may have favored mycorrhizal species, but it remains unclear what the driving factors were. Plant pathogens, however, are strongly specialized and dependent on a specific plant host.^83^^,^^84^ It can be assumed that those plant species were only present in St. John’s, which has a plant community distinct from the other two.^45^

For protists, we only found very few indicator species, around one-tenth of that of fungi, which were mostly bacterivores with some omnivores that both occupy higher trophic levels. This supports our findings, that related to earthworm invasion, protistan diversity showed similar patterns to that of bacteria, but that of protists were even more pronounced. This can be interpreted as a bottom-up effect where the diversity of prey fosters the diversity of the predator.^85^^,^^86^^,^^87^ Moreover, as stated before, organisms at higher trophic levels are more strongly affected by environmental stress than that at lower trophic levels.^72^^,^^88^

### Conclusions

Our study provides support for the negative effects of earthworm invasion on soil microbial communities. Compared to the other microbial kingdoms, fungal communities were relatively robust to earthworm invasion. Furthermore, communities were differently affected in different forests and soil depths. As a follow-up study of Ferlian et al. 2018,^22^ we explored microbial diversity, community composition, and abundances of microbial taxa and found that patterns across measures differed. Moreover, besides the taxonomic identity of the microorganisms, we further explored their functional identity to be able to broaden the existing knowledge on the ecological consequences of earthworm invasion via the effects on microbial communities. In particular, protists have been rarely considered in this context. Given the important role microorganisms play in soil,^89^^,^^90^ we assume that the effects we found can further cascade up to plants and animals and, thus, alter nutrient cycling and carbon storage in the system.^91^^,^^92^

### Limitations of the study

The taxonomic and functional assignments to DNA sequences rely on public databases, which still struggle with incomplete descriptions or even incorrect annotations.^93^ Moreover, compared to experimental studies, observational studies like ours generally do not allow for exploring causal relationships between predictor and response variables, such as earthworm invasion and microbial communities or environmental parameters in our case. However, part of our investigated forests and their invasion dynamics have been studied for three decades which partly allows for a careful distinction between the causes and consequences of the effects. Future studies can benefit from including more microbial functional traits in metagenomic approaches as well as plant community data to be able to draw a more comprehensive picture and gain a mechanistic understanding of the causes underlying soil biodiversity loss under global change.

## STAR★Methods

### Key resources table

**Table:** 

REAGENT or RESOURCE	SOURCE	IDENTIFIER
**Deposited data**		

Raw sequencing data of fungal/bacterial amplicons	This paper, National Center for Biotechnology Information (NCBI) Sequence Read Archives (SRA)	BioProject number PRJNA1001019
Raw sequencing data of protists	This paper, European Nucleotide Archive (ENA)	ERS2039495 (SAMEA104421553)
Environmental data	This paper, https://figshare.com	https://doi.org/10.6084/m9.figshare.24681054.v1
Statistical code	This paper, https://figshare.com	Published after acceptance

**Software and algorithms**		

R	R Core Team^56^	https://cran.r-project.org
QIIME 22020.2	Bolyen et al.^49^	https://qiime2.org

### Resource availability

#### Lead contact

Further information and requests for resources should be directed to and will be fulfilled by the lead contact, Olga Ferlian (olga.ferlian@idiv.de).

#### Materials availability

This study did not generate new unique reagents.

#### Data and code availability

• Microbial sequencing data have been deposited at the National Center for Biotechnology Information (NCBI) Sequence Read Archives (SRA; fungi and bacteria) and European Nucleotide Archive (ENA; protists) and are publicly available as of the date of publication. Accession numbers are listed in the key resources table. Environmental data have been deposited at the iDiv Data Repository and are publicly available as of the date of publication. The DOI is listed in the key resources table.
• All original code has been deposited at figshare.com and is publicly available as of the date of publication. The DOI is listed in the key resources table.
• Any additional information required to reanalyse the data reported in this paper is available from the lead contact upon request.

### Experimental model and study participant details

There were no experimental models involved in the study. Only soil samples were collected and immediately frozen for this study.

### Method details

#### Study site

We sampled three forest sites in northern North American forests in Canada and the USA, which have contrasting vegetation types and abiotic properties.^42^^,^^43^ We used information from literature on existing invasion fronts in these forests.^43^ The first and second forest are distinct boreal forests located in the Kananaskis Valley in the Rocky Mountains, southwest Alberta, Canada, north (51°02′N, 115°03′W, 1510 m a.s.l.; hereafter called Barrier North) and southwest of Barrier Lake (51°00′N, 115°04W, 1380 m a.s.l.; hereafter called Barrier South). The climate is continental with short dry summers and cold winters and a mean annual precipitation of 625 mm and temperature of 3.4°C. The soil pH is 6.1 and 5.8; the thickness of the organic soil layer is 7.3 and 5.5 cm; the soil nitrogen content is 0.86% and 0.48%; and the soil carbon content is 15.90% and 10.24%, respectively. The two forests are dominated by *Populus tremuloides* and *Populus balsamifera* trees interspersed with single *Picea glauca* trees on Orthic Grey Luvisol soils. The understorey vegetation is diverse with mostly *Rosa* and *Aster species*, *Viola canadensis*, *Epilobium angustifolium*, and *Delphinium glaucum*. The third forest is a temperate-boreal forest located near Saint John’s University, 120 km northwest of Minneapolis, Minnesota, USA (45°34′N, 94°23′W, 350 m a.s.l.; hereafter called St. John’s). The climate is humid continental and cold temperate and has a mean annual precipitation of 701 mm and temperature of 6.2°C. The soil pH is 4.9; the thickness of the organic layer is 1.6 cm; the soil nitrogen content is 0.35%; and the soil carbon content is 4.48%. This forest is dominated by *Acer saccharum* on Glossic Eutroboralf soil. The understorey vegetation is comparably sparse and comprises *Urtica dioica*, *Carex pennsylvanica*, and *Amphicarpaea bracteata*.

For three decades, several studies related to earthworm invasion have been conducted in one of the studied forests (Barrier North) and allow for a localisation of earthworm invasion fronts and their dynamics that served as the base of the present study (e.g.,^24^^,^^37^^,^^43^). Invasion front localisation in the other two forests was entirely based on digging for earthworms. Their distributions may be commonly patchy independently of invasion stage, which may confound invasion patterns and, thus, impede their detection. It is reported that no earthworms existed in these forests until 1984, and in 1992, only epigeic species (that typically colonize first) were found.^44^ In the subsequent years, endogeic and anecic species followed. Due to the proximity to fishing lakes and the direction of the invasion front movement, it is assumed that the earthworms were mainly introduced by anglers. In all forests, we conducted earthworm samplings to detect (St. John’s) and confirm (Barrier North and Barrier South) invasion fronts and the respective low and highly invaded areas. Barrier North has a south-facing slope with the low invaded area uphill and the highly invaded area downhill from the invasion front. Similarly, at Barrier South, the low and highly invaded areas are located uphill and downhill from the invasion front, respectively, but the slope is facing west. In Barrier North and Barrier South, the distance between the low and highly invaded area was 400 m; in St. John’s, the distance between the low and highly invaded area was 2.5 km. In St. John’s, the two areas were roughly on the same level and separated by a small lake. In the low invaded areas of each forest, earthworm abundances and biomasses equalled zero or were low comprising only epigeic earthworm species (see Figure S1 and Jochum et al.^45^ for earthworm abundances and biomasses, respectively).

#### Sampling

We set up ten plots of 1 × 1 m in the low and highly invaded area of each forest randomly in August 2016, respectively. Different samples were taken from this main plot including earthworm samples, following Jochum et al.^45^ Moreover, we took soil cores (three cores per plot pooled into one sample pre plot, 5 cm in diameter, separated into 0-5 and 5-10 cm depth) for measurements of carbon and nitrogen content, humus layers, litter weight, pH, and microbial biomass (see Jochum et al.^45^ for details on measurements). Each plot had an additional adjacent subplot of 0.3 × 1 m that was exclusively used for soil sampling and microbial analyses in this study to ensure that the soil had not been disturbed by other samplings. The minimum distance between plots was 15 m. For DNA sequencing analyses, we took four soil cores (diameter: 2 cm, depth: 10 cm) per plot under sterile conditions, subdivided them into 0-5 and 5-10 cm layers, homogenised them per plot and soil layer with a 2-mm sieve, and stored them in 15 ml plastic tubes on dry ice for transport. In the lab, samples were stored at -80°C until further processing.

#### DNA extraction and sequencing

Genomic DNA was extracted from soil microorganisms from soil samples using a PowerSoil DNA Isolation Kit (MO BIO Laboratories Inc., Carlsbad, California, USA) after the manufacturer’s protocol, with some modifications. We checked DNA yields with a NanoDrop ND-8000 spectrophotometer (Thermo Fisher Scientific, Dreieich, Germany) and stored the extracts at -20°C before further analyses. The DNA extracts were adjusted to 20 ng μl^−1^ template concentration, and the bacterial and fungal amplicon libraries were prepared as previously described.^46^^,^^47^ Briefly, the V4 region of the bacterial 16S rRNA gene and the fungal ITS2 fragment were amplified using primer pairs containing the Illumina adapter sequences. The amplicon libraries were purified and indexed. The indexed products were purified, quantified, and pooled equimolarly to a final concentration of 4 nM each for bacteria and fungi. The two libraries were then pooled proportionally to a final library and paired-end sequencing of 2 × 300 bp was performed on an Illumina MiSeq platform (Illumina, San Diego, CA, United States) using MiSeq Reagent kit v3. For protists, full information can be found in.^23^ Briefly, PCRs were conducted in two steps. In the first PCR, the forward primers S616F_Cerco and S616F_Eocer were mixed in the proportions of 80% and 20%, and used with the reverse primer S963R_Cerco.^48^ One μl of ten times diluted DNA were used as a template for the first PCR and 1 μl of the resulting amplicons were used as a template for a following semi-nested PCR. All PCRs were conducted twice to reduce the possible artificial dominance of few amplicons by PCR competition, and then pooled. A mock community with known species richness of diverse cultivated cercozoan taxa was run in parallel to assist the fine-tuning of the bioinformatics pipeline as described in.^48^ We pooled the samples and the mock community and proceeded for a single library preparation. Library preparation and paired-end MiSeq sequencing with the MiSeqv3 2 9 300 bp kit were carried out by the Cologne Center for Genomics (CCG). Raw sequencing data of the fungal and bacterial amplicons were deposited in the National Center for Biotechnology Information (NCBI) Sequence Read Archives (SRA) under the BioProject number PRJNA1001019. Sequencing data of the protists were deposited at the European Nucleotide Archive (ENA) under the accession number ERS2039495 (SAMEA104421553).

#### Bioinformatics

High-quality reads of fungi and bacteria were extracted from raw sequences generated by the Illumina MiSeq platform using the Quantitative Insights into Microbial Ecology – QIIME 22020.2 software.^49^ Following demultiplexing of forward and reverse reads based on index combinations, primer sequences were trimmed, followed by sequence denoising and grouping into amplicon sequence variants (ASVs) using cut-adapt^50^ (q2-cutadapt) and DADA2^51^ (via q2-dada2), respectively. The q2-feature-classifier^52^ was used to assign taxonomy to 16S bacterial ASVs, which was compared to the silva-132-99-515-806-nb-classifier^53^ using the classify-sklearn naive Bayes taxonomy classifier. The q2-ITSxpress Qiime2 plugin^54^ was used to analyse the fungal ITS dataset, with the ITS2 fungal sequences being detected and trimmed, followed by denoising and grouping into ASVs using DADA2.^51^ The q2-feature-classifier^52^ was used to classify fungal ITS ASVs using the classify-sklearn naive Bayes taxonomy classifier against the unite-ver8-99-classifier-04.02.2020.^55^ In both datasets, only ASVs assigned to a phylum level were filtered. For protists, the bioinformatic processing followed the descriptions by Dumack et al.^23^ and led to a clustering of operational taxonomic units (OTUs) at 97% sequence similarity, which were used for further statistical analyses.

### Quantification and statistical analysis

Subsequent analyses were based on rarefied datasets of bacteria (44,000 reads per sample), fungi (5324 reads per sample) and protists (5700 reads per sample) per sample. Statistical analyses were performed in R (version 4.2.2^56^).

We initially calculated ASV or OTU richness, Shannon diversity, evenness, and Simpson index using the R package *BiodiversityR* (2.15-2^57^). Boxplots, to visualise differences in microbial alpha diversity, were prepared. We used Wilcoxon rank sum exact tests (non-parametric tests) for evaluating whether earthworm invasion stage and/or soil depth affect microbial alpha diversity using the R package *vegan* (v 2.6-4^58^). We conducted ANOVAs to test the effects of earthworm invasion on environmental parameters as well as the interactions between earthworm invasion and environmental parameters on the microbial Shannon diversity index. To display shifts in microbial community compositions as affected by earthworm invasion stage and soil depth, non-metric multidimensional scaling (NMDS) was conducted and visualised based on Bray-Curtis dissimilarities considering relative ASV or OTU abundances using *vegan.*^58^ Permutational analysis of variance (perMANOVA), as implemented in *vegan*^58^*,* was applied to calculate the impact of earthworm invasion stage and soil depth on microbial communities. Microbial Bray-Curtis dissimilarities were further used to perform Mantel tests, evaluating potential correlations between pairwise bacterial, fungal, and protist community composition dissimilarities in the different invasion stages and soil depths. These comparisons were visualised as correlation plots prepared with the R package *corrplot* (v 0.92^59^).

Based on their relative abundances, the top ten bacterial and fungal families and top ten protist orders were plotted to display the impact of earthworm invasion stage and soil depth on microbial taxonomy. It has to be noted that more than those ten families/orders exist, but these represent only a small fraction of the community and were negligible for our analyses. Multi-level pattern analyses to identify bacterial, fungal, and protistan indicator species for either forest site, invasion stage, and soil depth were performed using the R package *indicspecies* (v 1.7.12^60^). Trophic groups of the top ten indicator microorganisms were assigned using FAPROTAX (v.1.2.6^61^) for bacteria, FungalTraits (v. 1.0^62^) for fungi, and Dumack et al.^23^ for protists.

## Supplemental information

Document S1. Figures S1–S4 and Tables S1 and S2

Table S3. Top 10 indicator species analysis Top 10 indicator species analysis with potentially indicative (if present, and based on overall association strength) (a) bacterial and (b) fungal ASVs and (c) protistan OTUs, respectively, for forest, invasion stage, and soil depths are shown. Statistical values: A - species only occurs in forests of this group, B - all forests of this group harbor this species, AS – overall association strength, p - significance value; NA - no functional annotation currently available. Relative abundances of ASVs and OTUs across all samples were considered. Indicator species analyses are outlined in the STAR Methods.
