# Supplementary material for: Invasive earthworms shift soil microbial community structure in northern North American forest ecosystems
Source: iScience. 2024 Jan 12;27(2):108889. doi: 10.1016/j.isci.2024.108889 (PMC10844042; doi:10.1016/j.isci.2024.108889)
Supplement: Document S1. Figures S1–S4 and Tables S1 and S2 [file mmc1.pdf]

iScience, Volume *27*

Supplemental information

Invasive earthworms shift soil microbial community structure in northern North American forest ecosystems

Olga Ferlian, Kezia Goldmann, Michael Bonkowski, Kenneth Dumack, Tesfaye Wubet, and Nico Eisenhauer

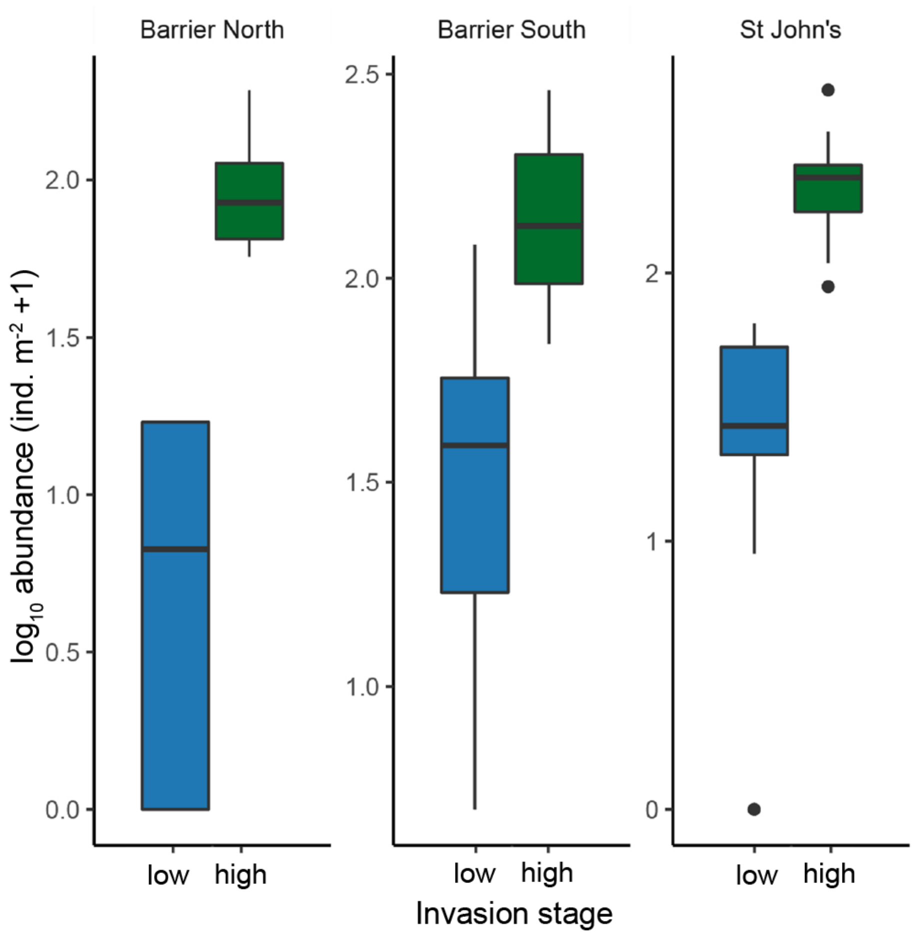

Figure S1 Earthworm abundances between low and high invasion stages, related to Figure 1-4.

Differences in earthworm abundances between low (in blue) and high (in green) invasion stages in the three northern North American forests. Values are log-transformed.

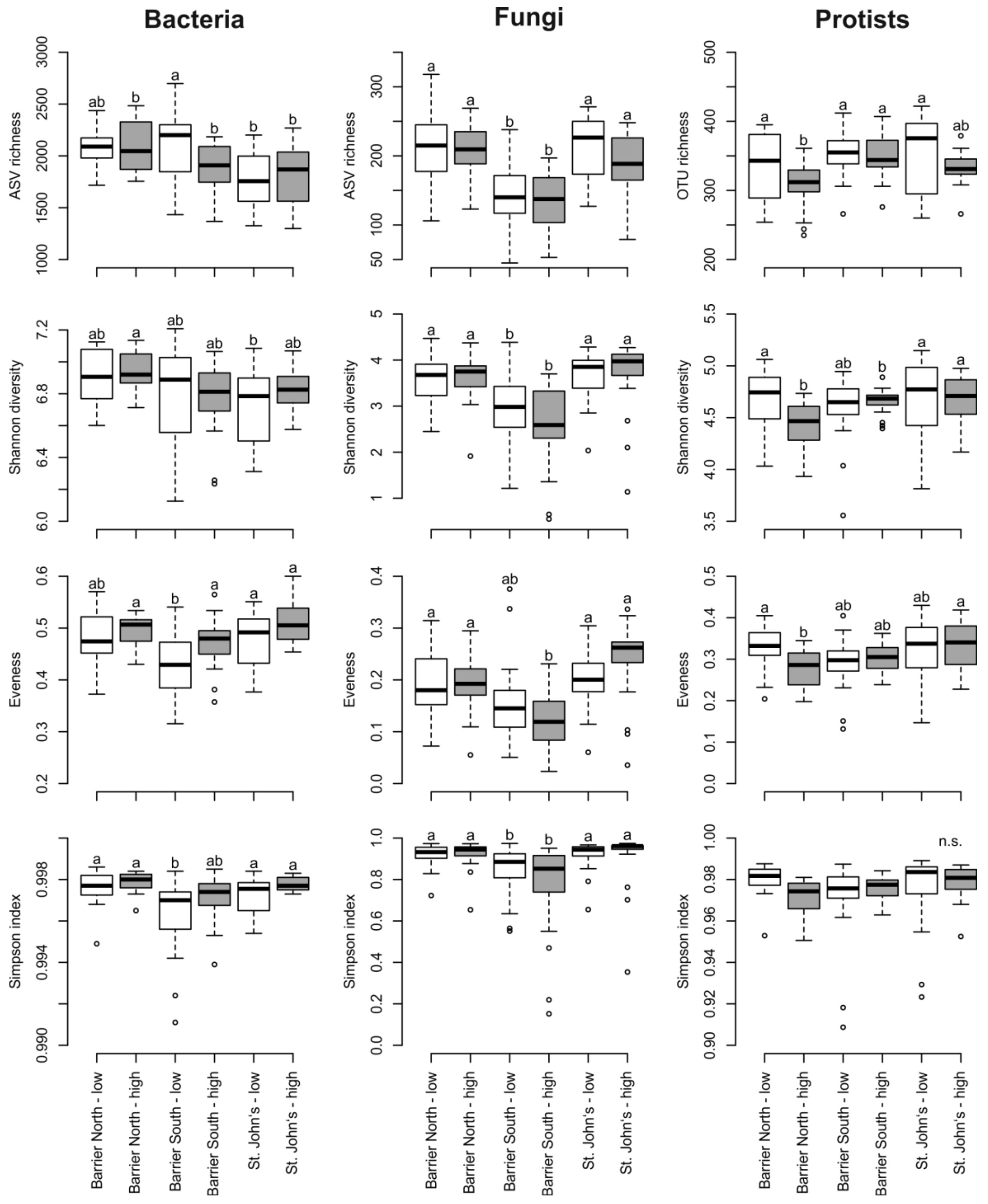

Figure S2 Total microbial ASV-richness, Shannon diversity, evenness and Simpson indices, related to Figure 1

Effects of forest and earthworm invasion stage for both soil depths on the four indices. Different letters above boxplots indicate significant differences (p≤0.05) according to Wilcoxon rank sum exact test; n.s. – no significant differences between considered predictor variables.

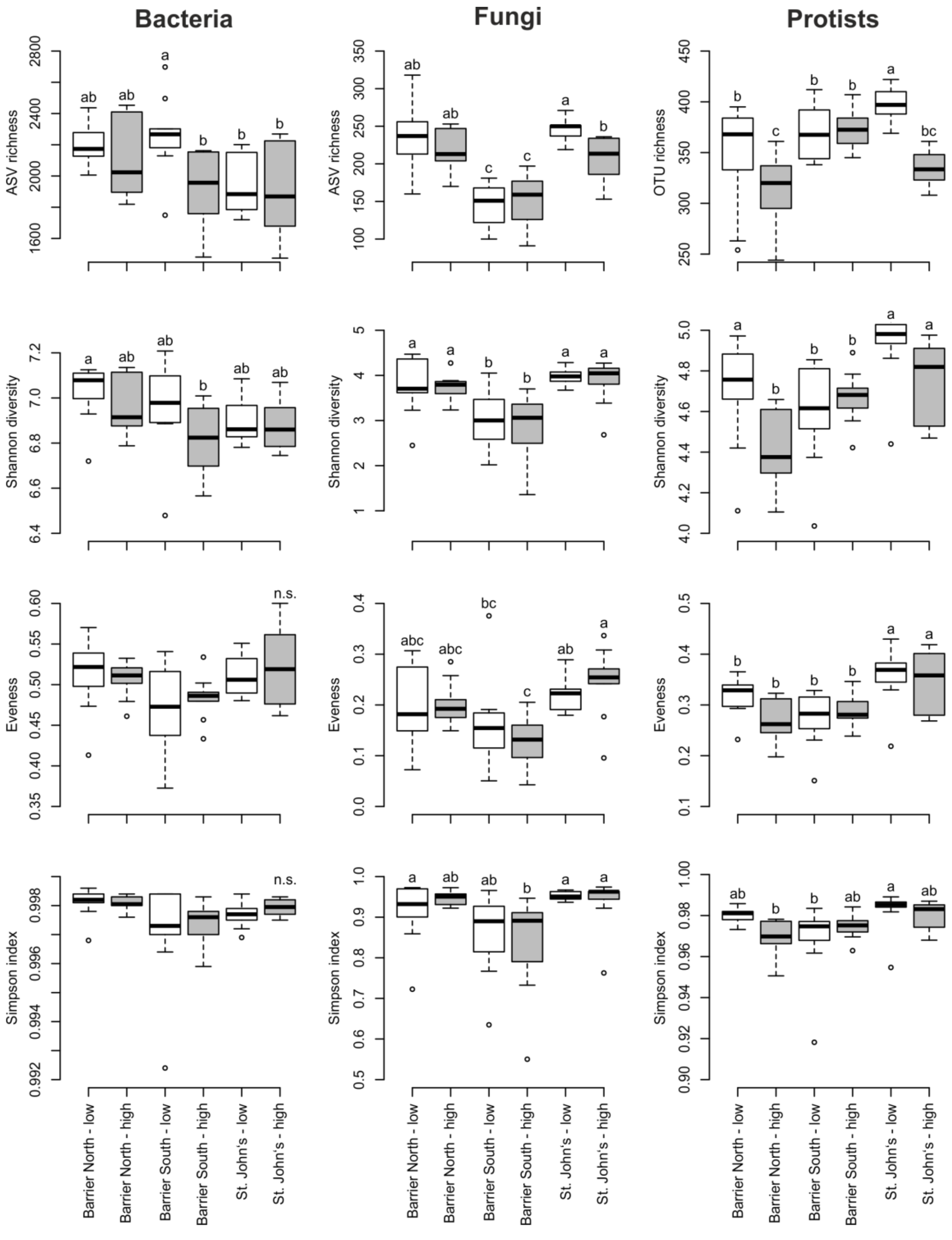

Figure S3 Microbial ASV-richness, Shannon diversity, evenness and Simpson indices for the upper soil layer, related to Figure 1

The effects of forest and earthworm invasion stage on the four indices for 0-5 cm soil depth. Different letters above boxplots indicate significant differences ($p \leq 0.05$) according to Wilcoxon rank sum exact test; n.s. – no significant differences between considered predictor variables.

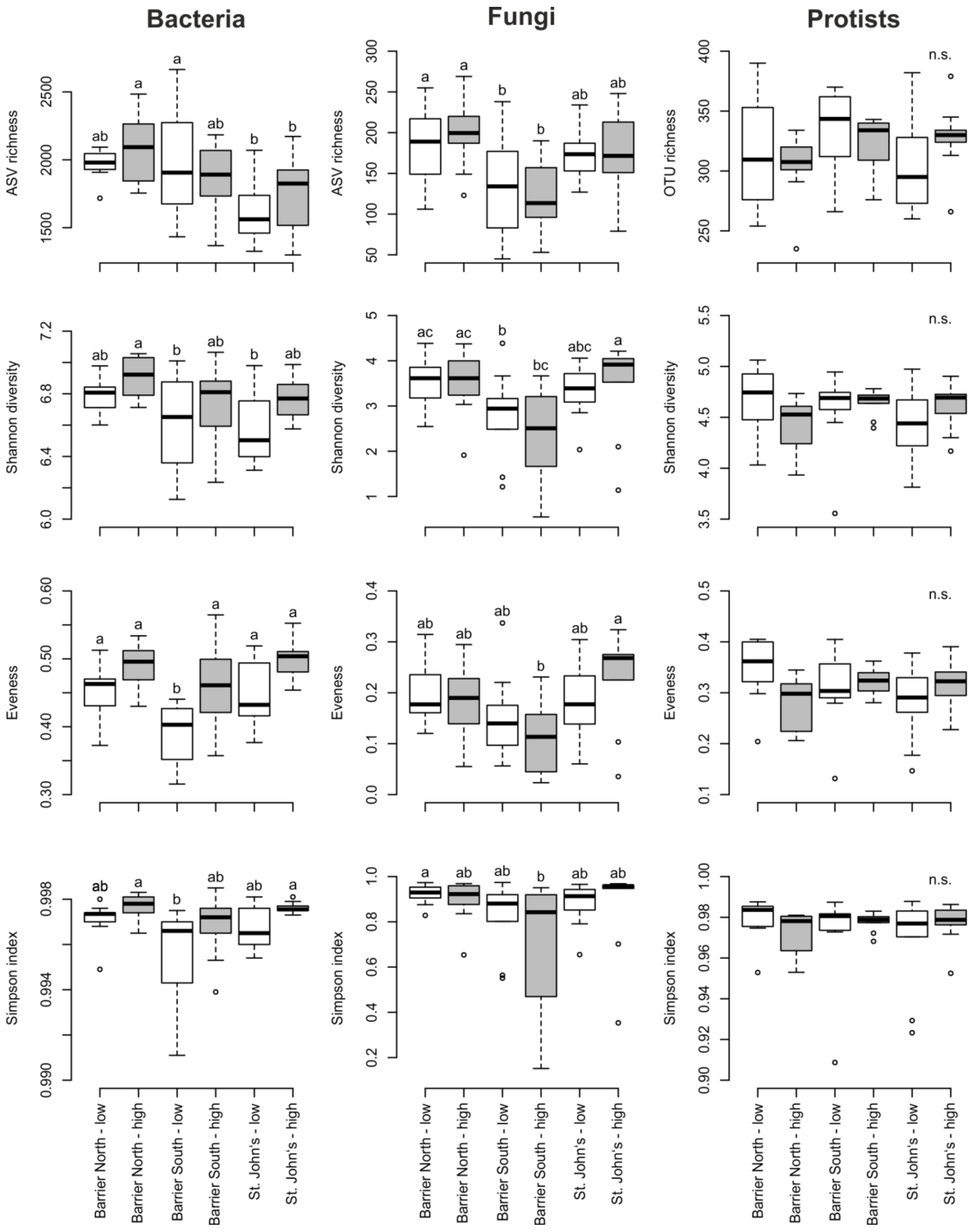

Figure S4 Microbial ASV-richness, Shannon diversity, evenness and Simpson indices for the lower soil layer, related to Figure 1

The effects of forest and earthworm invasion stage on the four indices for 5-10 cm soil depth. Different letters above boxplots indicate significant differences ($p \leq 0.05$) according to Wilcoxon rank sum exact test; n.s. – no significant differences between considered predictor variables.

Table S1 ANOVA summary table of the effects of earthworm invasion on environmental parameters, related to STAR Methods

Effects and means (±SD; in percent) of earthworm invasion on environmental parameters are shown for all three forests and soil depths. Values in bold indicate significant interactions. Asterisks show significance levels: * p ≤ 0.05; ** p ≤ 0.01; *** p ≤ 0.001). Arrows show direction of significant effect from low to highly invaded areas.

			low		high					
			Mean	SD	Mean	SD	$F_{1,18}$	p		
Barrier North		**Humus layer**	**8.45**	**2.49**	**6.20**	**1.30**	**6.43**	**0.02**	*	↓
		Litter weight	41.78	52.85	7.52	10.55	4.04	0.06		
		pH	6.45	0.28	6.24	0.32	2.45	0.14		
	0-5	**N concentration**	**2.00**	**0.66**	**0.66**	**0.26**	**35.90**	**<0.0001**	***	↓
		**C concentration**	**37.11**	**11.91**	**12.33**	**5.57**	**35.55**	**<0.0001**	***	↓
		**Microbial biomass**	**5459.61**	**1597.18**	**2219.99**	**765.86**	**33.45**	**<0.0001**	***	↓
	5-10	N concentration	0.46	0.25	0.31	0.10	3.31	0.09		
		C concentration	9.29	6.39	4.85	2.03	4.39	0.05		
		**Microbial biomass**	**1163.40**	**564.30**	**670.16**	**266.96**	**6.24**	**0.02**	*	↓
Barrier South		Humus layer	5.45	1.30	5.50	0.94	0.01	0.92		
		Litter weight	59.47	24.97	40.05	19.56	3.75	0.07		
		pH	6.21	0.50	5.91	0.41	2.15	0.16		
	0-5	**N concentration**	**1.13**	**0.31**	**0.42**	**0.09**	**37.72**	**<0.0001**	***	↓
		**C concentration**	**25.36**	**6.88**	**8.91**	**2.14**	**41.89**	**<0.0001**	***	↓
		**Microbial biomass**	**4533.78**	**923.09**	**1897.87**	**583.53**	**58.26**	**<0.0001**	***	↓
	5-10	**N concentration**	**0.24**	**0.05**	**0.12**	**0.04**	**26.64**	**<0.0001**	***	↓
		**C concentration**	**4.83**	**1.15**	**1.87**	**1.18**	**23.94**	**<0.001**	***	↓
		**Microbial biomass**	**836.53**	**212.61**	**339.57**	**114.54**	**42.34**	**<0.0001**	***	↓
St. John's		**Humus layer**	**2.90**	**0.88**	**0.25**	**0.35**	**78.76**	**<0.0001**	***	↓
		Litter weight	134.00	45.32	115.28	64.83	0.56	0.46		
		**pH**	**4.79**	**0.49**	**5.42**	**0.48**	**8.59**	**0.01**	**	↑
	0-5	**N concentration**	**0.75**	**0.23**	**0.28**	**0.05**	**40.58**	**<0.0001**	***	↓
		**C concentration**	**9.91**	**3.21**	**3.75**	**0.71**	**35.08**	**<0.0001**	***	↓
		**Microbial biomass**	**2241.89**	**1005.00**	**806.79**	**128.49**	**20.06**	**<0.001**	***	↓
	5-10	N concentration	0.17	0.08	0.21	0.06	1.53	0.23		
		C concentration	1.74	0.98	2.52	0.74	3.85	0.07		
		Microbial biomass	289.14	173.90	380.50	186.35	1.29	0.27		

Table S2 ANOVA summary table of the effects of the interactions between earthworm invasion and environmental parameters on microbial Shannon diversity, related to STAR Methods

Effects of the interactions between earthworm invasion and environmental parameters on microbial Shannon diversity are shown for all three microbial taxa across the three forests and soil depths. Values in bold indicate significant interactions. Asterisks show significance levels: * p ≤ 0.05; ** p ≤ 0.01).

			Barrier North			Barrier South			St. John's	
			$F_{1,18}$	p		$F_{1,18}$	p		$F_{1,18}$	p
Bacteria	0-5	Invasion*Humus layer	0.320	0.592		0.007	0.941		0.094	0.769
		Invasion*Litter weight	1.008	0.354		0.333	0.604		0.293	0.608
		Invasion*pH	1.959	0.211		0.039	0.855		0.001	0.974
		Invasion*C concentration	0.120	0.741		0.589	0.499		0.400	0.550
		Invasion*N concentration	0.558	0.483		0.247	0.653		0.023	0.884
		Invasion*Microbial biomass	0.325	0.589		0.022	0.892		0.465	0.521
	5-10	Invasion*Humus layer	0.626	0.459		0.027	0.896		0.084	0.783
		Invasion*Litter weight	**9.626**	**0.021**	*	0.551	0.593		0.109	0.755
		Invasion*pH	1.862	0.221		0.383	0.647		0.062	0.813
		Invasion*C concentration	**17.151**	**0.006**	**	0.593	0.582		0.000	0.983
		Invasion*N concentration	1.582	0.255		1.165	0.476		0.826	0.405
		Invasion*Microbial biomass	1.728	0.237		0.144	0.769		0.740	0.429
Fungi	0-5	Invasion*Humus layer	1.563	0.258		9.676	0.053		0.102	0.761
		Invasion*Litter weight	0.102	0.761		**14.373**	**0.032**	*	2.350	0.176
		Invasion*pH	0.001	0.979		9.379	0.055		0.098	0.765
		Invasion*C concentration	4.071	0.090		0.037	0.859		0.013	0.912
		Invasion*N concentration	0.402	0.550		**59.916**	**0.004**	**	1.628	0.249
		Invasion*Microbial biomass	1.173	0.320		**25.589**	**0.015**	*	0.047	0.835
	5-10	Invasion*Humus layer	1.548	0.260		30.970	0.113		2.329	0.188
		Invasion*Litter weight	0.086	0.779		115.527	0.059		0.656	0.455
		Invasion*pH	0.764	0.416		1.580	0.428		0.009	0.926
		Invasion*C concentration	1.613	0.251		**315.826**	**0.036**	*	0.676	0.448
		Invasion*N concentration	0.350	0.576		**247.836**	**0.040**	*	0.679	0.448
		Invasion*Microbial biomass	0.473	0.517		44.292	0.095		0.440	0.537
Protists	0-5	Invasion*Humus layer	4.233	0.085		**10.667**	**0.047**	*	0.013	0.912
		Invasion*Litter weight	0.688	0.439		1.914	0.261		1.230	0.310
		Invasion*pH	0.643	0.453		0.955	0.401		1.214	0.313
		Invasion*C concentration	1.670	0.244		0.230	0.665		0.247	0.637
		Invasion*N concentration	0.119	0.742		0.091	0.783		0.023	0.885
		Invasion*Microbial biomass	0.044	0.841		6.028	0.091		0.521	0.498
	5-10	Invasion*Humus layer	0.055	0.823		0.632	0.572		0.641	0.460
		Invasion*Litter weight	0.029	0.870		1.354	0.452		2.737	0.159
		Invasion*pH	0.244	0.639		0.164	0.755		0.066	0.808
		Invasion*C concentration	0.685	0.439		20.104	0.140		0.185	0.685
		Invasion*N concentration	1.474	0.270		4.303	0.286		4.402	0.090
		Invasion*Microbial biomass	0.098	0.764		6.936	0.231		0.039	0.851
